# Specificity of DNA ADP-Ribosylation Reversal by NADARs

**DOI:** 10.3390/toxins16050208

**Published:** 2024-04-28

**Authors:** Bara Cihlova, Yang Lu, Andreja Mikoč, Marion Schuller, Ivan Ahel

**Affiliations:** 1 Sir William Dunn School of Pathology, University of Oxford, Oxford OX1 3RE, UK; bara.cihlova@univ.ox.ac.uk (B.C.); yang.lu@exeter.ox.ac.uk (Y.L.); 2 Division of Molecular Biology, Ruđer Bošković Institute, 10000 Zagreb, Croatia; mikoc@irb.hr

**Keywords:** ADP-ribosylation, toxin–antitoxin system, DNA modification, PARP, DNA damage, YbiA, DarTG

## Abstract

Recent discoveries establish DNA and RNA as bona fide substrates for ADP-ribosylation. NADAR (“NAD- and ADP-ribose”-associated) enzymes reverse guanine ADP-ribosylation and serve as antitoxins in the DarT-NADAR operon. Although NADARs are widespread across prokaryotes, eukaryotes, and viruses, their specificity and broader physiological roles remain poorly understood. Using phylogenetic and biochemical analyses, we further explore de-ADP-ribosylation activity and antitoxin functions of NADAR domains. We demonstrate that different subfamilies of NADAR proteins from representative *E. coli* strains and an *E. coli*-infecting phage retain biochemical activity while displaying specificity in providing protection from toxic guanine ADP-ribosylation in cells. Furthermore, we identify a myxobacterial enzyme within the YbiA subfamily that functions as an antitoxin for its associated DarT-unrelated ART toxin, which we termed YarT, thus presenting a hitherto uncharacterised ART-YbiA toxin–antitoxin pair. Our studies contribute to the burgeoning field of DNA ADP-ribosylation, supporting its physiological relevance within and beyond bacterial toxin–antitoxin systems. Notably, the specificity and confinement of NADARs to non-mammals infer their potential as highly specific targets for antimicrobial drugs with minimal off-target effects.

## 1. Introduction

ADP-ribosylation (ADPr) holds regulatory significance in diverse biological processes and is ubiquitous across organisms in all kingdoms of life [1,2,3,4]. In mammals, the most extensively studied case is the role of ADPr signalling in the DNA damage response by PARP enzymes. However, ADPr controls many other physiological aspects, including immunity, chromatin structure, apoptosis, and development [1,5,6]. Traditionally, ADPr in higher organisms has focused on protein substrates, while nucleic acids were only recently identified as targets [7,8,9,10,11,12,13,14]. In bacteria, ADPr is used for both precise regulation of endogenous signalling, but even more commonly for attack and defence strategies due to its extreme toxicity [2,15,16,17,18,19]. While bacterial ADPr is beginning to be unravelled, its wider significance and occurrence in higher organisms remain largely unknown, partly due to the lack of suitable methodologies [7].

The first system discovered to engage in DNA ADPr is a family of toxins known as pierisins that cause irreversible modifications of guanines [20,21]. Several members of this family have been characterised in mechanistic detail, including Pierisin-1 in larvae of cabbage butterfly or Scabin and ScARP, secreted from the plant pathogen *Streptomyces scabies* and *Streptomyces coelicolor*, respectively [22,23,24]. Recently, RNA has also emerged as a potential target for irreversible toxic ADPr by Rhs toxins released via several type VI secretion systems (T6SS) into competitor bacteria. The Tre23 effector in *Photorhabdus laumondii* modifies 23S ribosomal RNA [25,26], while the RhsP2 effector in *Pseudomonas aeruginosa* has been shown to modify dsRNAs and tRNAs on 2′OH groups of RNA [27]. Although inherently irreversible, both systems incorporate scavenging immunity proteins—RhsI2 for RhsP2 and Tri23 for Tre23—that protect the secreting cells from the ADPr activities of the toxin.

In contrast, reversible systems of nucleic acid ADPr were established by the toxin–antitoxin (TA) system of DarT2 and DarG, which remains the best characterised system to date [18,28]. Mono-ADPr by DarT2 is sequence-specific, targeting a thymidine base in single-stranded DNA (ssDNA) [18]. DarT2 modification is counteracted by the antitoxin DarG [18], which relies on the catalytically active ADP-ribosylhydrolase of the macrodomain type [29]. This imparts precision to the reversible DarT2 modification, distinct from the pierisins that display variable substrate preferences and a relaxed sequence specificity [30]. The modification has been shown to behave as a site of DNA damage in enteropathogenic *Escherichia coli* (*E. coli*), stalling bacterial replication and growth, possibly to promote persistence amidst antibiotic treatment [31]. Likewise, DarT2 targets replication origins and controls growth in *Mycobacterium tuberculosis* [28], which characteristically causes persistent and challenging-to-treat infections. Accordingly, reversal of this modification by DarG has been shown to be required for bacterial growth [31]. Modifications by DarT2 also play a role in antiphage defence [31,32]. Most recently, a system with a homologous DarT toxin component termed “DarTG1”, i.e., DarT1-NADAR (distinguished from the DarT2-DarG system), was described in bacteria [33]. DarT1 targets guanosine bases in ssDNA without a defined sequence specificity, while the ADP-ribosyl hydrolase domain, NADAR (“NAD- and ADP-ribose”-associated enzymes [34]), seems specifically engaged in removing guanosine–ADPr, alleviating DarT1 toxicity. The NADAR domain is evolutionary unrelated to the antitoxin macrodomain that is found in the DarT2 system. The NADAR superfamily is characterised by the YbiA fold, comprising five core helices flanked by two small sheets [33]. The fold is shared by two subfamilies, the “YbiA family” and the “BC4488 family” [34]. The distinguishing feature for the families is a conserved charged residue in the last strand, shown to be contributing to the catalytic centre [33]. This residue is a glutamate in the YbiA family, an aspartate in the BC4488 NADAR family, and a histidine in phage NADARs—also termed gp30.3 proteins. Furthermore, the association with the ADP-ribosyltransferase (ART) DarT seems to be exclusive to the BC4488 family of NADARs [33,34].

**Figure 1:**
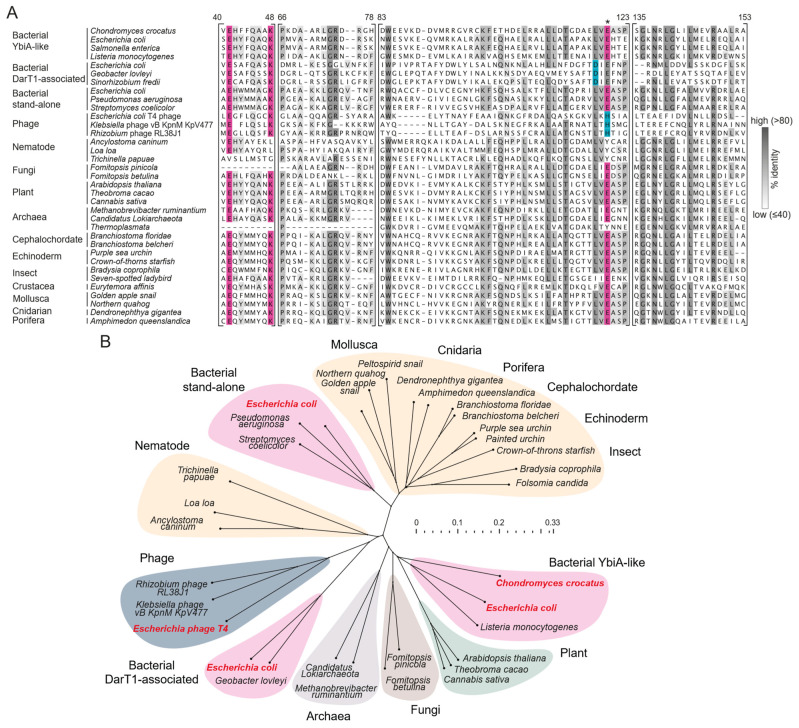
**Catalytically competent NADAR members are widespread in nature.** (**A**) Multiple sequence alignments of selected NADAR superfamily members across groups of organisms. Catalytic residues are highlighted in pink. The distinguishing catalytic aspartate and histidine residues of DarT1-associated and phage NADARs, resp., are highlighted in blue; these residues are thought to correspond to the catalytic glutamate that is present in other NADARs, marked with an asterisk. Residue numbers refer to *Chondromyces crocatus* NADAR. (**B**) Phylogenetic tree constructed using multiple sequence alignments of NADARs (Table S1), highlighting bacterial (pink), animal (orange), and other groups by colour. Species in bold red represent proteins selected for biochemical analysis in this study.

The functional significance of NADAR domains is poorly understood, but they have been implicated in at least four diverse processes. NADAR proteins in bacteria and plants, annotated as *N*-glycosidases, were suggested to function as regulatory enzymes in the initial two steps of riboflavin biosynthesis to detoxify excess reactive intermediates [35]. Additionally, the *ybiA* gene, encoding a member of the YbiA family of NADARs, was shown to be involved in swarming motility in the K-12 strain of *E. coli* [36], and this phenotype can be induced by a phage-expressed *ybiA* during the lysogenic cycle [37]. In *Caenorhabditis elegans*, the nuclear protein SNA-3 with three NADAR domains has been implicated in the process of trans-splicing, a nematode-specific mechanism of mRNA splicing [38]. Lastly, the antitoxin function of NADAR domains in DarT1 operons, as described earlier, represents another recognised role for these enzymes [33]. Notably, most bacterial NADARs exist as stand-alone domains, and some genomes host several NADAR/YbiA domains, suggesting the divergence of this protein family into distinct non-redundant functions.

In this study, we examined bacterial and phage NADAR proteins and found a shared ability to hydrolyse ADPr on guanosine bases. Genes encoding these proteins are found in different genetic contexts, and despite retaining a conserved in vitro activity, we observed pronounced specificity in providing protective effects against toxic guanosine ADP-ribosylated DNA in vivo. Moreover, the genetic context of one of the *ybiA*-like genes revealed a so far uncharacterised toxic ART domain belonging to the H-Y-[E/D/Q] clade, unveiling the first ART-YbiA toxin–antitoxin system. Collectively, our data reveal a lively evolution of NADAR domains and guanosine–ADPr, indicating higher specificity and a more widespread occurrence than previously acknowledged.

## 2. Results

### 2.1. E. coli Hosts Multiple NADAR Domains with Different Substrate Specificities

Recently, we solved crystal structures of two representative NADAR proteins from a thermophilic bacterial species and a eukaryotic oomycete species and discovered their ADP-ribosyl hydrolase activity on guanosine–ADPr [33]. Our analyses indicate that NADARs can be identified in organisms across all kingdoms of life, including viruses, with high conservation of catalytically relevant residues (Figure 1A,B). In metazoans, NADARs are found in numerous species, such as arthropods, nematodes, mollusks, echinoderms, porifera, and cnidarians. Notably, NADAR domains are also present in *Brachiostoma*, which belongs to the most basal subphylum of vertebrates, Cephalochordata.

**Figure 2:**
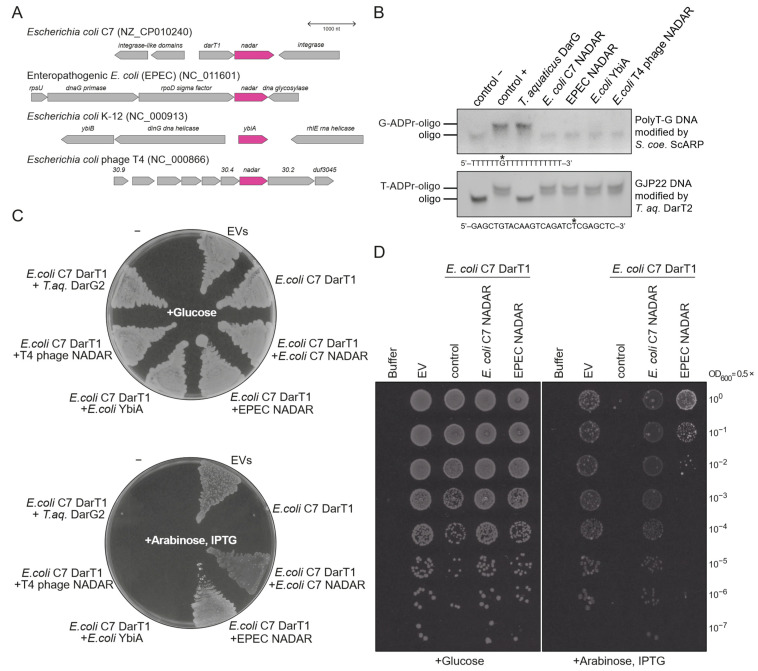
**NADAR family members found in *E. coli* hosts and phages have distinct specificities.** (**A**) Genomic environments of NADARs that occur in *E. coli* and an *E. coli*-infecting phage. (**B**) In vitro activity assay using ADP-ribosylated DNA substrates at indicated positions (asterisks) and purified recombinant NADARs. (**C**) In vivo toxicity assay of DarT1 toxin, complemented by NADARs encoded by the *E. coli* strains and *E*. *coli*-infecting phage tested in (**B**). Whereas glucose represses expression, arabinose induces expression of the DarT1 toxin, and IPTG induces expression of potentially complementing antitoxins. Only *E. coli* C7 and EPEC NADARs provide complementary antitoxin activity. (**D**) Titration of the same conditions plated in (**C**), showing efficiency in rescuing from DarT1-induced cytotoxicity effects. Representative for three biologically independent experiments. Empty vectors (EVs), i.e., empty pBAD33 and pET28a vectors.

Hence, we aimed to explore the presence of guanosine–ADPr activity in other members of the NADAR superfamily. Our initial focus was on the *E. coli* system. Based on the sampled strains, it appears that there are typically no more than two NADAR members within a single *E. coli* strain. In total, we identified four distinct NADAR domains across different strains, indicating a dynamic evolution of NADARs, with multiple instances of horizontal gene transfer. These NADAR subtypes are found in different genomic contexts (Figure 2A). Besides the characterised DarT1-associated NADAR antitoxin, found in strains such as C7 and ETEC (O36:H5), we detected a NADAR subtype integrated into a conserved operon, along with genes encoding three key proteins involved in DNA replication, transcription, and protein translation—the σ^70^ sigma factor, DNA primase, and ribosomal protein S21 [39,40]. This NADAR corresponds to a “stand-alone” bacterial NADAR, i.e., it is not fused to functional domains in a single polypeptide or characterised with functionally associated enzymes (Figure 1B), and it is present in several important pathogenic strains, including DEC (O55:H6), EPEC (O127:H6), and STEC (O157:H7). In addition, we identified “K-12 YbiA-like” bacterial NADARs in many *E. coli* strains, consistently found inside a LexA-controlled operon induced by DNA damage [41]. Apart from *ybiA*, the operon contains a DNA helicase *dinG*, an RNA helicase *rhlE*, and *ybiB*, which was predicted to have a DNA-binding and phosphoribosyltransferase activity [41,42]. Lastly, we noted NADAR proteins (gp30.3) in a number of *E. coli* phages, including the tailed T4 phage [43].

We proceeded to test all four NADAR subtypes—DarT1-linked, σ^70^-linked, YbiA, and phage-encoded—using two different substrates: an established PolyT-G substrate modified with ADP-ribose on a single guanosine by *S. coelicolor* ScARP, and a model substrate with thymidine-ADPr catalysed by *Thermus aquaticus (T. aq.)* DarT2 [18,22]. While all tested NADAR enzymes showed an inability to reverse thymidine ADPr, unlike DarG, we did observe hydrolytic activity of all NADAR/YbiA proteins on guanosine ADPr (Figure 2B). This aligns with the observed conservation of proposed catalytic residues in all these enzymes [33] (Figure 1A). To test whether this de-ADPr activity is sufficient to rescue from DarT1 toxicity in cells, we conducted sequential co-transformation of cells with different NADAR proteins, along with *E. coli* C7 DarT1—an established guanine-specific ADP-ribosyl transferase [33]. Plating the doubly transformed cells on plates with arabinose, which allowed for the expression of DarT1, induced toxicity, unless a sufficiently specific NADAR antitoxin was present. Interestingly, YbiA and phage NADAR proteins were unable to provide DarT1-protective antitoxin activity in vivo (Figure 2C), which was similar to the control DarG2, which possesses thymidine-ADPr specificity. EPEC σ^70^-linked NADAR showed an ability to rescue from guanine ADPr to some extent, yet additional serial dilution experiments of transformed cells confirmed that it is less effective than DarT1-linked NADAR, as expected (Figure 2D). Therefore, our data strongly suggest that, despite conserved in vitro activity on model substrates, NADAR domains achieve high levels of substrate specificity, which is relevant in a physiological setup.

### 2.2. Myxobacterial YbiA-like Proteins Function as Antitoxins for the ART Toxin YarT

**Figure 3:**
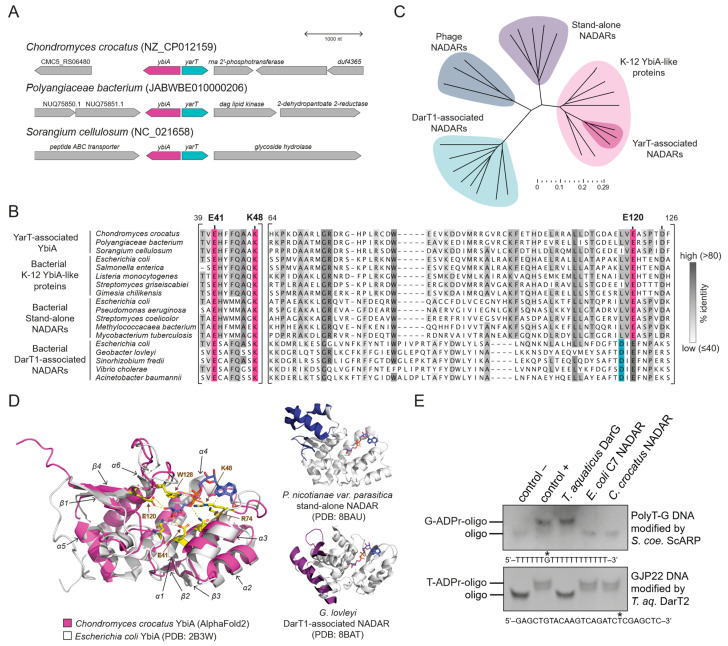
**Myxobacterial YbiA proteins retain conserved K-12 YbiA-like structure and catalytic activity.** (**A**) Operon environments of YbiA-YarT toxin–antitoxin pairs in three myxobacterial species. (**B**) Multiple sequence alignment comparing YarT-associated YbiA to other bacterial NADARs. Catalytic residues are shown in pink and blue, as in Figure 1. (**C**) Phylogenetic tree showing clustering of YarT-associated YbiA. Compared are only phage and bacterial NADARs from species shown in (**A**). NCBI accession numbers are summarised in Table S2. (**D**) **Left**: structural overlay of an AlphaFold2 model of YarT-associated YbiA from *C. crocatus* to the crystal structure of *E. coli* K-12 YbiA. **Right**: crystal structures of NADARs, showing the core K-12 YbiA-like domain (white) and characteristic N-terminal extensions (coloured). (**E**) In vitro activity assay using ADP-ribosylated DNA substrates at indicated positions (asterisks) and purified recombinant NADARs. Representative for three biologically independent experiments.

To better understand the functions of the YbiA family of NADARs (Figure 1), we searched for uncharacterised operons encoding bacterial K-12 YbiA-like proteins beyond *E. coli* strains. Our investigation led to the identification of a group of myxobacteria encoding a YbiA-like protein upstream of an uncharacterised protein (Figure 3A). The subsequent sequence alignment and phylogenetic analysis confirmed that the myxobacterial proteins cluster together with members of the YbiA family and retain characteristic catalytic residues (Figure 3B,C). An AlphaFold2 model of the myxobacterial YbiA-like proteins predicts a core YbiA domain without any fold extension (Figure 3D), in contrast to the characterised NADARs from *Phytophthora nicotianae* or *Geobacter lovleyi* [33]. The structural divergence from DarT1-linked NADAR is also reflected in the notably better overlay of the model with crystal structures of *E. coli* K-12 YbiA and the stand-alone *P. nicotianae* NADAR (RMSD values of 1.15 and 0.95, resp.) compared to the DarT1-linked NADAR from *E. coli* (RMSD of 4.88). To test whether the uncharacterised YbiAs retain guanosine ADPr hydrolytic activity, we purified myxobacterial *C. crocatus* YbiA and tested its activity in vitro. Indeed, *C. crocatus* YbiA could reverse guanosine ADPr catalysed by ScARP, similarly to *E. coli* C7 NADAR (Figure 3E). Once again, we confirmed conserved de-ADPr activity among the members of the NADAR enzyme superfamily.

**Figure 4:**
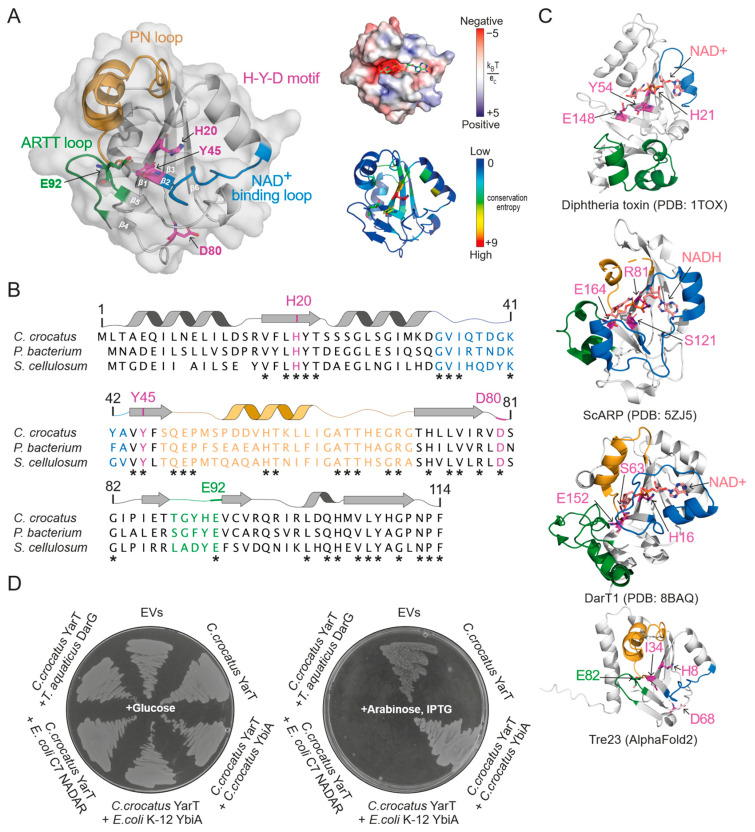
**YarT is an ART toxin complemented by an YbiA antitoxin.** (**A**) **Left**: Cartoon representation of *Chondromyces crocatus* YarT, as predicted by AlphaFold2, with ART-characteristic features highlighted and discussed in the main text. **Right top**: surface electrostatic potential, calculated using APBS, with docked NAD^+^ in the predicted active site. **Right bottom**: B factors, determined by conservation and mapped on the YarT AlphaFold2 model by ConservFold. (**B**) Primary and secondary structure of YarT proteins from three myxobacterial species, showing conserved residues (asterisks), and ART-characteristic features coloured as in the Cartoon model. (**C**) Crystal structures of selected ARTs with ART-characteristic features highlighted as in (**A**,**B**), with motif residues shown in purple. Glutamate residues shown in green in YarT and Tre23 are in the corresponding structural positions to the characterised catalytic glutamate in ARTD (DT) and ARTC (ScARP) family members, as well as in DarT, where E152 was modelled in to the original solved structure of an E152A mutant. (**D**) In vivo toxicity assay showing the bacteriostatic effects induced by *C. crocatus* YarT expression. Complementation with *C. crocatus* YbiA rescues the toxic phenotype of YarT, demonstrating that the YarT and YbiA behave as a toxin–antitoxin pair. Representative for three biologically independent experiments. Empty vectors (EVs), i.e., empty pBAD33 and pET28a vectors.

We next turned our attention to the hypothetical protein encoded downstream to YbiA in the operons of three myxobacterial species. Inspection of its AlphaFold2 model revealed a conserved ADP-ribosyltransferase (ART) fold inside a small globular protein (Figure 4A,B). Intrigued by the possible connection between a so far uncharacterised ART and YbiA, we designated this protein as “YarT” (YbiA-associated ADP-ribosyl transferase). YarT is predicted to have an openly accessible NAD^+^-binding cleft, with no discernible substrate preference based on the surface electrostatic potential (Figure 4A). ARTs are typically categorised into two families: ARTD (diphtheria toxin-like) and ARTC (cholera toxin-like) [2]. YarT is predicted to harbour the characteristic H-Y-[E/D/Q] motif of the ARTD family. Other ARTD features shared by YarT include (1) a six-stranded β-sheet core formed by two distinct units of three strands each, (2) the NAD^+^-binding loop between β1 and β2, and (2) the ARTT loop inserted between β4 and β5 that has been implicated in substrate recognition and binding [44]. However, YarT combines these elements in a much more simplified fold, with no extensions and much shorter loops, compared to diphtheria toxin (a prototypic ARTD) (Figure 4C).

Interestingly, YarT clusters within the HYD1 subclade of bacterial ARTs alongside the toxin domain of “Rearrangement hot spot” (Rhs) proteins (Figure S1) [45]. Rhs proteins are highly polymorphic components of type VI secretion systems (T6SS), featuring an N-terminal domain targeting the secretion machinery, a central domain with conserved motifs, and a highly variable C-terminal toxin domain that is subjected to cleavage upon secretion [46]. YarT resembles the C-termini of certain Rhs proteins (Rhs^CT^), notably exemplified by Tre23 from *Photorhabdus laumondii*, known to ADP-ribosylate RNA at an uncharacterised site [25,26]. Our FoldSeek search for structural homologues of YarT further confirmed its similarity to Rhs^CT^ toxins, showing the very high similarity of the secondary structure arrangements between the AlphaFold2 models of the exemplary *P. laumondii* Tre23 toxin domain and *C. crocatus* YarT (Figure 4C). Of note, we noticed conservation of an H-Y-E motif in YarT (Figure 4A), despite clustering into the HYD1 subclade of the ARTs, which was defined purely by a bioinformatic analysis [45]. The conserved glutamate (E92) is in a position reminiscent to catalytic glutamates found in well-characterised ARTs, including the ARTD prototype DT and the ARTC member ScARP. Interestingly, we also identified such glutamate residue in a structurally similar position in Tre23 which, however, remains uncharacterised regarding a contribution to the catalytic function of this toxin [25,26]. Yet, with its position close to the NAM-ribose of NAD^+^ being carried by the loop region next to β5, the glutamate E92 may present a catalytically relevant residue of YarT. This discrepancy between a HYD classification and a catalytically more plausible HYE motif may motivate us to re-think and more closely investigate the definition of poorly characterised ART subclades and their motifs with regards to their catalytic residues.

Due to the extreme toxicity and insolubility of the YarT, we were unable to examine its activity in vitro. Thus, we examined whether the YarT ART exhibits activity in vivo by causing bacteriostatic effects in *E. coli*. Indeed, cells that had been transformed with YarT failed to form colonies upon inducing protein expression with arabinose (Figure 4D), indicating biochemical activity within the cellular context. Co-transformation with the YarT-associated *C. crocatus* YbiA protein was sufficient to reverse the toxic phenotype (Figure 4D). This rescue demonstrates that YarT and YbiA function as a toxin–antitoxin pair, confirming that the observed toxicity is a result of biochemical activity and not, for instance, protein expression artefacts. The observation that *C. crocatus* YbiA is an active hydrolase of guanine ADPr that can reverse YarT toxicity implies that YarT likely operates within cells to ADP-ribosylate guanosine bases. Conversely, the absence of rescue from DarG, which specifically targets thymidine–ADP-ribose linkages, suggests that YarT is unlikely to be involved in thymidine ADPr (Figure 4D).

On the other hand, both *E. coli* K-12 YbiA and C7 NADAR, which are guanosine-specific ADP-ribosyl hydrolases, failed to alleviate YarT-induced toxicity (Figure 4D). This aligns with the specificity seen in genetically linked toxin–antitoxin pairs. Specifically, although *E. coli* C7 NADAR effectively counteracts the toxicity of *E. coli* C7 DarT1 (Figure 2C,D), it fails to rescue the toxicity of YarT (Figure 4D). Similarly, YbiA from *E. coli* K-12 does not act as an antitoxin for either DarT1 or YarT. However, a structurally similar YbiA from *C. crocatus* efficiently reverses *C. crocatus* YarT toxicity, as anticipated from a genetically defined toxin–antitoxin pair. Hence, our findings underscore the notable specificity for ADP-ribosylated substrates and/or other mechanisms that confer specificity within these toxin–antitoxin systems, even among closely related NADAR and YbiA enzymes.

## 3. Discussion

DNA and RNA recently emerged as substrates for ADPr [7]. Reversible ADPr of nucleic acids has since gained recognition as a common signalling and warfare strategy in bacteria. Macrodomains and NADARs are key players in these processes, functioning as antitoxin components, self-standing hydrolases, or defence modules [18,33,47]. What remains unclear is the physiological roles of bacterial NADARs outside DarT operons, as well as NADARs found across eukaryotes, including animals.

In this study, we identify several subtypes of NADAR domains and showcase their activities on ADP-ribosylated DNA model substrates and in an *E. coli* model system. The essential residues for de-ADPr of DNA are conserved across the NADAR superfamily, and, indeed, all characterised *E. coli* host or phage NADARs retain catalytic activity. Yet, this hydrolytic activity is likely to be associated with functions in different physiological contexts, guided by the substrate specificity of the respective NADAR protein. Thus, while NADARs that are genetically linked to DarT1 efficiently protect the host from toxic guanosine base ADPr products in its DNA, we found that NADARs in other genetic contexts are unable to act efficiently as antitoxins against DarT1 in the bacterial host. In several *E. coli* strains, we found stand-alone NADARs associated with a conserved operon containing a σ^70^ sigma factor (*rpoD*), a DNA primase (*dnaG*), and a ribosomal S21 protein (*rpsU*) [40]. These genes are involved in the initiation of transcription, replication, and translation, respectively. NADAR may thus be coregulated with the three central processes involving nucleic acids. This raises questions about whether NADARs actively participate in transcription, replication, or translation, or if they act as safeguards against exogenous ARTs that target these essential processes. The former mechanism would indicate a yet unrecognised importance of reversible ADPr of nucleic acids during normal physiology. On the other hand, the latter role was previously suggested for macrodomain-containing proteins induced by DNA damage in *Streptomyces* [19,47]. Beyond their role in reversing endogenous DarT signalling, which was shown to affect replication [28,31], it is conceivable that certain bacteria possess specialised NADARs to counteract the obstructing effects of ADPr by exogenous (e.g., phages) or other endogenous ARTs during replication and gene expression.

In the case of phages, the presence of NADAR domains suggests a potential defence strategy against host ARTs, providing specific protection for phage DNA from DNA-modifying bacterial toxins. Both bacterial and phage DNA carry modifications distinguishing self from invading genetic material, likely influencing hydrolytic activity efficiency and the specificity of the respective NADAR domain. The molecular basis determining the substrate specificity of NADARs remains to be explained. YbiA fold extensions, which vary among NADARs, could be considered for the basis of specificity. However, proteins from the YbiA family lack such extensions, existing essentially as “naked” YbiA domains. We show that specificity is retained even among YbiA proteins, since *E. coli* YbiA fails to rescue YarT toxicity, unlike *C. crocatus* YbiA. The importance of extensions is therefore not universal, and equally important for substrate specificity might be the residues on the surface and at the catalytic site, possibly driving substrate recognition, coordination, and hydrolytic processing. In addition, we ought to consider the potential for direct interactions between NADARs and their target toxins. In fact, DarG was found to directly bind DarT to disable its ADPr activity [18,48]. Gaining insights into this concept is crucial for understanding the functions of the NADAR superfamily and predicting the roles of yet uncharacterised NADAR proteins.

The present study also led to the identification of a hitherto undescribed YarT-YbiA toxin–antitoxin system, which may play a role in phage defence, similarly to DarTG. YbiA proteins form a subfamily of the NADAR superfamily, distinguished by a glutamate catalytic residue in the last β strand as opposed to an aspartate or histidine [34]. The observed YbiA hydrolytic activity on ADP-ribosylated guanosine bases in DNA suggests that YarT toxicity may result from ADPr activity on guanosines, akin to DarT1. Yet, the more simplistic make-up of YarT, especially in the substrate-binding region compared to other transferases like DarTs, implies a potentially less restrained substrate targeting. YarT might induce toxicity through ADPr on a wider range of substrates, either by lacking sequence specificity (unlike DarT2) or by targeting non-DNA molecules that allow for *N*-glycosidic ADP-ribose linkages. Despite repeated attempts to purify a recombinant version of YarT, we were unable to recover isolated protein, likely due to unbearable toxicity and/or folding problems. Future studies will be necessary to elucidate the target specificity of YarT. Additional considerations include the potential requirement of accessory factors for YarT and NADARs to attain specificity for their respective substrates (DNA, RNA, protein, or small molecules), modification sites (e.g., DNA ends versus different DNA bases), and ADPr type (e.g., mono-ADPr, poly-ADPr, and RNAylation). In its stand-alone form, YarT is limited to a few myxobacterial species with a non-conserved genomic environment, yet it preserves all functional ART features, including an NAD^+^-binding loop and a conserved glutamate (E92). In myxobacteria, YbiAs may act as antitoxin modules, safeguarding against stand-alone YarT toxins that cannot be secreted, unlike the structurally similar Rhs toxins such as Tre23 (Figure 4C) [46] and therefore demand a unique form of tight regulation.

In summary, we confirm a conserved de-ADPr activity on guanosine bases in different NADAR family members, found within *E. coli* strains and an *E. coli*-infecting phage. An intriguing finding was that the de-ADPr activity alone did not confer antitoxin activity against DarT1 modification in vivo, indicating additional mechanisms governing substrate specificity and causing a need for species- or even strain-specific antitoxins. Discovery of a novel YarT-YbiA toxin–antitoxin pair suggests that different NADAR superfamily members, including YbiA subfamily members, can take roles as antitoxins. Targeting NADAR antitoxin with small molecule inhibitors could induce strong bacteriostatic effects due to the unregulated activity of their respective toxins, from which the host cannot recover. Thus, NADARs may emerge as promising drug targets with high selectivity for the specific pathogen, as suggested for the DarTG system [28]. In addition, our analyses highlight the apparent absence of NADARs in higher vertebrates, including mammals and humans, suggesting that drugs targeting NADARs might have very low off-target effects. We anticipate that elucidating both the biochemical mechanisms and physiological roles of nucleic acid ADPr in lower organisms will pave the path for antimicrobial drug discovery and improve our understanding of this phenomenon in higher organisms.

## 4. Materials and Methods

### 4.1. Materials, Reagents, and Chemicals

High-fidelity DNA polymerase Phusion, Gibson Assembly, and Gateway cloning reagents were obtained from New England Biolabs (Hitchin, UK) and Thermo Scientific (Waltham, MA, USA). DNA primers and ssDNA substrates (Supplementary Table S3) were synthesised by Thermo Scientific (US). All remaining chemicals were purchased from Sigma (Darmstadt, Germany), unless stated otherwise.

### 4.2. DNA Cloning

The full-length *Chondromyces crocatus* NADAR (residues 1–156), *Chondromyces crocatus* YarT (residues 1–114), EPEC NADAR (residues: 1–187), *E. coli* T4 phage NADAR (gp30.3; residues: 1–208), and *E. coli* YbiA (residues: 1–160) genes were synthesised and cloned into a pET28a vector by GenScript. The full-length *Chondromyces crocatus* YarT (residues 1–114) was subsequently re-cloned into a pBAD33 vector. All constructs are summarised in Supplementary Table S5.

### 4.3. Recombinant Protein Expression and Purification

*E. coli* C7 NADAR, *T. aq*. DarT2 and *T. aq*. DarG, and *S. coelicolor* ScARP (SCO5461) were produced as previously described [19,33]. For production of EPEC NADAR, *E. coli* T4 phage NADAR, and *E. coli* YbiA protein, an *E. coli* Rosetta strain BL21(DE3) was transformed with the corresponding constructs and grown at 37 °C in LB media supplemented with 50 μg/mL of kanamycin. Upon reaching an OD_600nm_ of 0.6, the temperature was reduced to 18 °C, and 0.5 mM IPTG was added for overnight (O/N) protein expression. The pelleted cells were resuspended in lysis buffer (50 mM HEPES (pH 7.5), 500 mM NaCl, 5% glycerol, 20 mM imidazole, 0.5 mM TCEP, and complete EDTA-free protease inhibitors (Roche) (Basel, Switzerland)) and stored at −20 °C. For purification, cells were thawed on ice, supplemented with benzonase and lysozyme, and lysed by eight cycles of 1 min sonication at 15 microns of amplitude with a 1 min pause between each cycle. Purification was performed by immobilised metal affinity chromatography (IMAC) using Ni-Sepharose resin (GE Healthcare (Chicago, IL, USA)), eluting in a lysis buffer with 40–500 mM imidazole and 1 M NaCl. Eluted proteins were dialysed O/N into a buffer containing 50 mM HEPES (pH 7.5), 250 mM NaCl, 5% glycerol, and 0.5 mM TCEP. All proteins were analysed by SDS-PAGE, concentrated in a 10,000 Da spin concentrator (Millipore (Burlington, VT, USA)), and flash-frozen in liquid nitrogen for storage at −80 °C. Protein concentrations were determined by measuring absorption at 280 nm with the DS-11 FX nanodrop (DeNovix (Wilmington, DE, USA)).

### 4.4. Toxicity Assays

BL21(DE3) cells were transformed sequentially with kanamycin-resistant plasmids and chloramphenicol-resistant plasmids, resp., according to an adapted protocol by Chung et al. [49]. Following the first transformation, five colonies were picked and grown to the early exponential phase (OD_600nm_ of 0.3–0.4) in the presence of 0.8% (*w*/*v*) glucose and 50 µg/mL kanamycin. Pelleted cells were resuspended in one-tenth of their original volume in ice-cold TSS buffer, consisting of LB medium with 10% (*w*/*v*) PEG 3350, 5% (*v*/*v*) DMSO, and 50 mM MgCl_2_ and transformed for the second time. Doubly transformed cells were selected O/N on LB agar with 0.8% (*w*/*v*) glucose, 25 µg/mL chloramphenicol, and 50 µg/mL kanamycin. Five colonies were picked and grown up in LB medium with 0.8% (*w*/*v*) glucose and appropriate antibiotics until reaching OD_600nm_ of ~0.5. All samples were adjusted to an OD_600nm_ of 0.5, followed by the preparation of 1:10 dilution series, which were spotted onto LB agar plates. These plates contained specific antibiotics for selection and either 0.8% (*w*/*v*) glucose or 0.8% (*w*/*v*) arabinose/50 µM IPTG to either suppress or induce recombinant protein expression, resp. The effects on bacterial growth were assessed after an O/N incubation at 37 °C. The results of all experiments are representative of a minimum of two biological replicates.

### 4.5. Gel-Shift ADP-Ribosylation Activity Assays

Oligo ADP-ribosylation experiments were conducted in a buffer solution composed of 50 mM Tris-Cl at pH 8.0, 50 mM NaCl, and 5 mM ETDA. The reactions were carried out in a 10 µL reaction volume at 37 °C, lasting 30 min for *T. aquaticus* DarT2 and 60 min for *S. coelicolor* ScARP. In the case of *T. aquaticus* DarT2, 1 µM of the enzyme was exposed to 3 µM oligonucleotides, using an excess of *β*-NAD^+^ (500 µM). For *S. coelicolor* ScARP, 0.1 µM of the protein was added to 10 µM oligonucleotides, along with an excess of *β*-NAD^+^ (3 mM). The ADP-ribosylation reaction was stopped for oligo de-modification by hydrolases by heating the samples for 15 min at 95 °C. Subsequently, the samples were either incubated with buffer for control purposes or with 1 μM of the specified hydrolase at 37 °C for 30 min.

The reaction products were separated on denaturing polyacrylamide gels in 1 × TBE buffer after the addition of 10 µL urea loading dye (10 mM TRIS pH 8.0, 10 mM EDTA, 4 M urea), followed by a 3 min incubation at 95 °C. Then, 10 µL of the treated samples were loaded onto the gel, and the oligos were visualised under UV light (340 nm) after staining with ethidium bromide or SYBR Gold nucleic acid stain (Thermo Scientific (US)). The results of all experiments are representative of a minimum of three biological replicates.

### 4.6. Structural and Phylogenetic Analyses

Multiple sequence alignments were created using Jalview v2 [50] and MAFFT7 [51]. Predictions of protein structures were generated using ColabFold [52], and structural homology searches were performed using FoldSeek [53]. Structural visualisation and analysis were conducted in PyMOL v2.5.3 (Schrödinger LLC (New York, NY, USA)). Conservation entropy was determined using ConservFold [54].

Phylogenetic relationships in Figure 1 and Figure 3 were inferred using the Neighbour-Joining (NJ) method [55], and confidence levels were estimated using 1000 replicates of the Bootstrap method [56] in SplitsTree CE 6.0.0_alpha [57]. The scale represents the percentage of trees where the associated taxa clustered together. Distance matrices were obtained using the Hamming method [58], and the cyclic split network was computed by NeighborNet [59]. Corresponding NCBI accession numbers are provided in Supplementary Tables S1 and S2.

The evolutionary relationships in Figure S1 were inferred by using the Maximum Likelihood method and Whelan and Goldman model [60] in MEGA X [61]. The tree with the highest log likelihood (−10,515.87) is shown. The percentage of trees in which the associated taxa clustered together is represented by the scale. Initial trees for the heuristic search were obtained automatically by applying NJ and BioNJ algorithms to a matrix of pairwise distances, estimated using the JTT model. A discrete Gamma distribution was used to model evolutionary rate differences among sites (5 categories (+G, parameter = 4.2708)). The rate variation model allowed for some sites to be evolutionarily invariable ([+I], 0.31% sites). This analysis involved 28 protein sequences with a total of 327 positions in the final dataset. Corresponding NCBI accession numbers are provided in Supplementary Table S3.

## Data Availability

The data presented in this study are available in this article or Supplementary Materials.

## Supplementary Materials

The following supporting information can be downloaded at https://www.mdpi.com/article/10.3390/toxins16050208/s1: Figure S1: Phylogenetic tree of bacterial ART toxins.; Table S1: NCBI accession IDs related to Figure 1; Table S2: NCBI accession IDs related to Figure 3; Table S3: NCBI accession IDs related to Figure S1; Table S4: Oligonucleotides used in this study; Table S5: Strains and plasmids used in this study. Reference [62] is cited in the supplementary materials.
