# Supplementary material for: Specificity of DNA ADP-Ribosylation Reversal by NADARs
Source: Toxins (Basel). 2024 Apr 28;16(5):208. doi: 10.3390/toxins16050208 (PMC11125620; doi:10.3390/toxins16050208)
Supplement: Supplementary file 1 [file toxins-16-00208-s001.zip › toxins-2935093-supplementary.pdf]

Specificity of DNA ADP-Ribosylation Reversal by NADARs

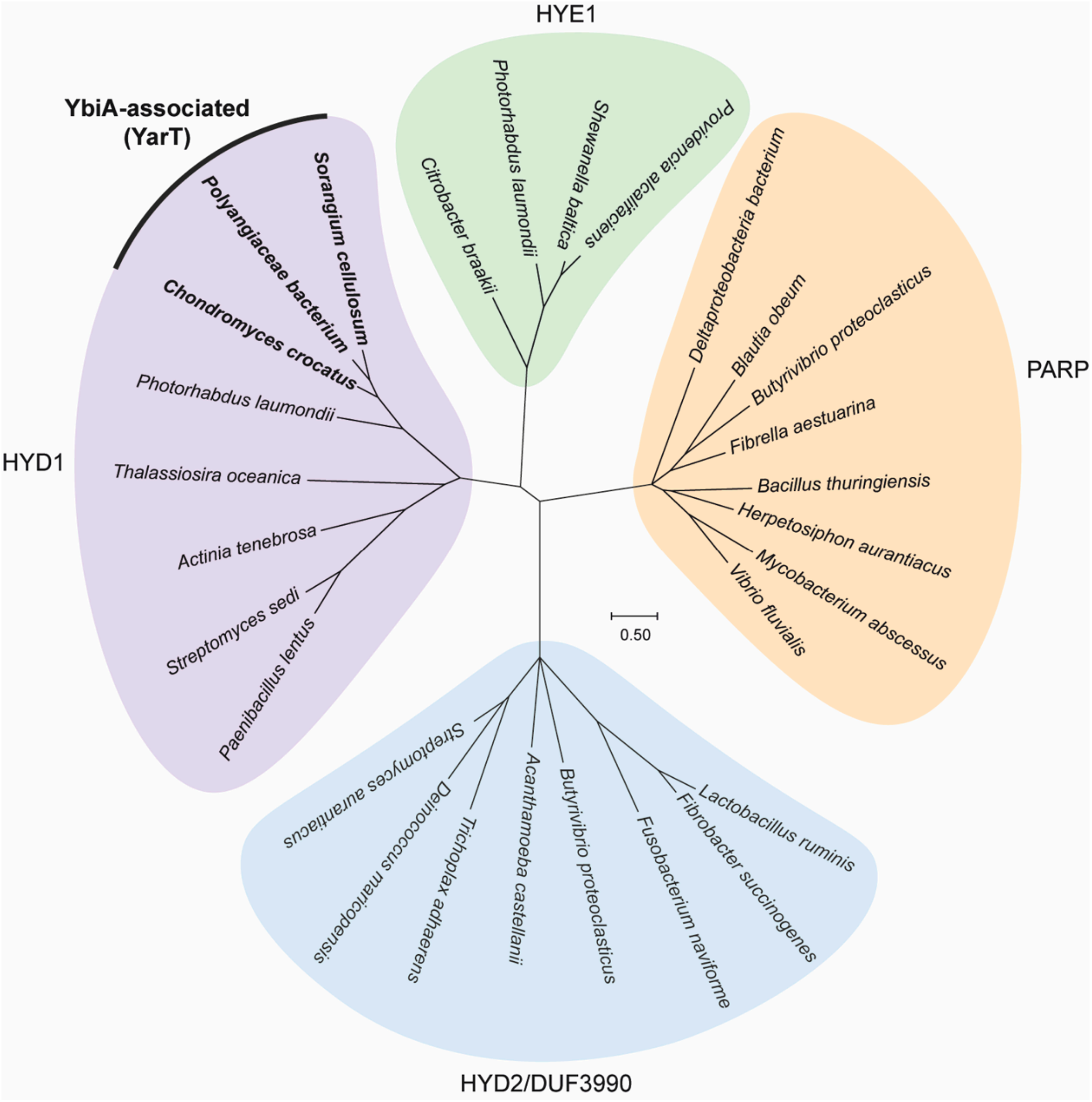

Figure S1. Phylogenetic tree of bacterial ART toxins. The YbiA-associated YarT ARTs cluster within the HYD1 subfamily. NCBI accession IDs are provided in Table S3.

Table S1. NCBI accession IDs related to Figure 1.

Species	Accession number
**Bacterial YbiA-like**	
Chondromyces crocatus	WP_179955513.1
Escherichia coli	WP_001145126.1
Salmonella enterica	WP_140040215.1
Listeria monocytogenes	HAA3934926.1
**Bacterial DarT-associated**	
Escherichia coli	WP_032219797.1
Geobacter lovleyi	WP_012470628.1
Sinorhizobium fredii	WP_014330845.1
**Bacterial stand-alone**	
Escherichia coli	WP_001183948.1
Pseudomonas aeruginosa	WP_116806626.1
Streptomyces coelicolor	BDD75137.1
**Phage**	
Escherichia coli T4 phage	NP_049816.1
Klebsiella phage vB KpnM KpV477	YP_009288818.1
Rhizobium phage RL38J1	QGZ13929.1
**Fungi**	
Fomitopsis pinicola	EPS93933.1
Fomitopsis betulina	KAI0715550.1
**Plant**	
Arabidopsis thaliana	VYS59636.1
Theobroma cacao	EOY09810.1
Cannabic sativa	KAF4360218.1
**Archaea**	
Methanobrevibacter ruminantium	WP_012956765.1
Candidatus Lokiarchaeota	MBD3226909.1
Thermoplasmata	MBE6519942.1
**Nematode**	
Ancylostoma caninum	RCN47812.1
Loa loa	XP_003139559.1
Trichinella papuae	KRZ69666.1
**Cephalochordate**	
Brachiostoma floridae	XP_035698547
Brachiostoma belcheri	XP_019631451
**Echinoderm**	
Purple sea urchin	XP_793312
Painted urchin	XP_054764016
Crown-of-thorns starfish	XP_022087852
**Insect**	
Bradysia coprophila	XP_037037289
Seven-spotted ladybird	XP_044764571

Folsomia candida	XP_021960032
**Crustacea**	
Eurytemora affinis	XP_023334262
**Mollusca**	
Golden apple snail	XP_025111217
Northern quahog	XP_045159950
Peltospirid snail	XP_041358894
**Cnidaria**	
Dendronephtya gigantea	XP_028391651
**Porifera**	
Amphimedon queenslandica	XP_019853895

Table S2. NCBI accession IDs related to Figure 3.

Species	Accession number
**YarT-associated YbiA proteins**	
Chondromyces crocatus	WP_179955513.1
Polyangiaceae bacterium	NUQ75852.1
Sorangium cellulosum	WP_020736121.1
**YbiA proteins**	
Escherichia coli	WP_001145126.1
Salmonella enterica	WP_140040215.1
Listeria monocytogenes	HAA3934926.1
Streptomyces griseiscabiei	WP_086756638.1
Gimesia chilikensis	QDT22974.1
**DarT-associated NADAR proteins**	
Escherichia coli	WP_001183948.1
Geobacter lovleyi	WP_012470628.1
Sinorhizobium fredii	WP_014330845.1
Vibrio cholerae	WP_172778105.1
Achinetobacter baumannii	WP_001129309.1
**Stand-alone NADAR proteins**	
Escherichia coli	WP_032219797.1
Pseudomonas aeruginosa	WP_116806626.1
Streptomyces coelicolor	BDD75137.1
Methylococcacaea bacterium	NOQ36470.1
Mycobacterium tuberculosis	CNF61934.1
**Phage NADAR proteins**	
Escherichia coli T4 phage	NP_049816.1
Klebsiella phage vB KpnM KpV477	YP_009288818.1
Rhizobium phage RL38J1	QGZ13929.1

Table S3. NCBI accession IDs related to Figure S1.

Species	Accession number
**HYD1**	
Chondromyces crocatus	WP_050429593.1
Polyangiaceae bacterium	NUQ75853 .1
Sorangium cellulosum	WP_020736122 .1
Paenibacillus lentus	AZK48993.1
Streptomyces sedi	WP_139640642.1
Photorhabdus laumondii	WP_011144745.1
Thalassiosira oceanica	EJK58845.1
Actinia tenebrosa	XP_031550746.1
**HYD2**	
Deinococcus maricopensis	WP_148234928.1
Streptomyces aurantiacus	WP_190851997.1
Fusobacterium naviforme	KAB0576670.1
**DUF3990**	
Lactobacillus ruminis	WP_014073068.1
Butyrivibrio proteoclasticus	WP_013281589.1
Fibrobacter succinogenes	WP_014546709.1
Trichoplax adhaerens	XP_002114834.1
Acanthamoeba castellanii	XP_004358258.1
**HYE1**	
Shewanella baltica	WP_006083171.1
Citrobacter braakii	WP_149330609.1
Photorhabdus laumondii	WP_011145957.1
Providencia alcalifaciens	WP_207910800.1
**PARP**	
Bacillus thuringiensis	EEM25277.1
Fibrella aestuarina	WP_041257497.1
Mycobacterium abscessus	WP_271960408.1
Vibrio fluvialis	WP_020332344.1
Deltaproteobacteria bacterium	MBI1947919.1
Herpetosiphon aurantiacus	ABX07394.1
Blautia obeum	WP_055060497.1
Butyrivibrio proteoclasticus	WP_013282825.1

Table S4. Oligonucleotides used in this study.

Oligo	Sequence (5'-3')	Purpose
PolyT-G	TTTTTTGTTTTTTTTTTT	ADP-ribosylation activity assay
DarT-27mer-3	CACGACACGAGCAGGCATGTCCACGTG	ADP-ribosylation activity assay
GJ-P22	GAGCTGTACAAGTCAGATCTCGAGCTC	ADP-ribosylation activity assay
Protein X_fwd	ctagcgaattcgagctcatgGGCAGCAGCCATCATCATC	Cloning of *C. crocatus*
Protein X_rev	ccgccaaaacagccaagcttTTAGAACGGGTTCGGGCC	Protein X into

		pBAD33 by Gibson Assembly
pBAD33_fwd	AAGCTTGGCTGTTTTGGC	Vector amplification for Gibson Assembly
pBAD33_rev	CATGAGCTCGAATTCGCTAG	

Table S5. Strains and plasmids used in this study.

Strain	Description	Source
DH5α	*huA2 a(argF-lacZ)U169 phoA glnV44 a80a(lacZ)M15 gyrA96 recA1 relA1 endA1 thi-1 hsdR17*	NEB
BL21 (DE3)	*fhuA2 [lon] ompT gal (λ DE3) [dcm] ΔhsdS λ DE3 = λ sBamHIo ΔEcoRI-B int::(lacI::PlacUV5::T7 gene1) i21 Δnin5*	NEB
Rosetta™ BL21 (DE3)	*F-ompT hsdSB(rB- mB-) gal dcm (DE3) pRARE (cam^R)*	Novagen
**Plasmid**		
pET28a	Medium copy plasmid containing the IPTG-inducible promoter; kan^R	Novagen
pBAD33	Medium copy plasmid with an arabinose-inducible promoter; cam^R	Guzman et al., 1995 [62]
pBAD33_Taq_*darT2*	pBAD33 carrying *T. aquaticus darT* full-length; cam^R	Jankevicius et al., 2016 [18]
pET28_Taq_*darG*_macro	pET28a carrying *T. aquaticus darG* macrodomain (aa 1-155); kan^R	Jankevicius et al., 2016 [18]
pET28_SC_*SCO5461*	pET28a carrying *S. coelicolor scarp (SCO5461)* full-length; kan^R	Lalić, J. et al., 2016 [19]
pET28_EPEC_*nadar*	pET28a carrying EPEC *nadar* full-length; kan^R	This study
pET28_Ecoli_*nadar*	pET28a carrying *E. coli* C7 *nadar* full-length; kan^R	Schuller et al., 2023 [33]
pET28_T4_*nadar*	pET28a carrying *E. coli* T4 phage (gp30.3) *nadar* full-length; kan^R	This study
pET_Ecoli_*ybia*	pET28a carrying *E. coli* K-12 *ybiA* full-length; kan^R	This study
pBAD33_Ecoli_*darT1*	pBAD33 carrying *E. coli* C7 *darT1* full-length; cam^R	Schuller et al., 2023 [33]
pBAD33_Ccroc_*nadar*	pBAD33 carrying *C. crocatus nadar* full-length; cam^R	This study
pBAD33_Ccroc_*yarT*	pBAD33 carrying *C. crocatus yarT* full-length; cam^R	This study
